# Sequencing of two mitochondrial genomes of endangered form of the Sevan trout *Salmo ischchan aestivalis*

**DOI:** 10.1080/23802359.2018.1462120

**Published:** 2018-04-23

**Authors:** Artem V. Nedoluzhko, Evgeniy Simonov, Sergey M. Rastorguev, Eugenia S. Boulygina, Fedor S. Sharko, Svetlana V. Tsygankova, Van Quan Nguyen, Bardukh K. Gabrielyan, Haikaz R. Roubenyan, Boris A. Levin

**Affiliations:** a National Research Centre “Kurchatov Institute”, Moscow, Russia;; b Laboratory of Fish Ecology, Papanin Institute for Biology of Inland Waters, Russian Academy of Sciences, Borok, Yaroslavl Region, Russia;; c Laboratory of Forest Genomics, Siberian Federal University, Krasnoyarsk, Russia;; d Institute of Bioengineering, Research Center of Biotechnology of the Russian Academy of Sciences, Moscow, Russia;; e Institute of Marine Environment and Resources, Vietnam Academy of Science and Technology, Ha Noi, Vietnam;; f Scientific Center of Zoology and Hydroecology, National Academy of Sciences of Republic of Armenia, Yerevan, Armenia;; g Department of Biology, Cherepovets State University, Cherepovets, Vologda Region, Russia

**Keywords:** Mitochondrial genome, Endangered species, *Salmo ischchan aestivalis*, Sevan trout, Illumina sequencing

## Abstract

The two complete mitochondrial genomes of endangered form of the Sevan trout *Salmo ischchan aestivalis* are published in this paper. The mitochondrial DNA (mtDNA) is 16,677 base pairs (bp) in length and contained 13 protein-coding genes, 2 rRNA genes, and 22 tRNA genes. The overall base composition of the genome in descending order was 29.4% – C, 27.9% – A, 26.0% – T, 16.7% – G, without a significant AT bias of 53.9%.

## Main text

DNA samples of the Sevan trout, Endangered subspecies *Salmo ischchan aestivalis*, were obtained from two individuals: Li4 individual was collected from local aquaculture farm near Djermuk town on 2012 (39.7305 N and 45.5950 E) and Li30 was collected from the natural population, the Makenis River, tributary of Lake Sevan, on 6 May 1974 (40.1771 N and 45.5841 E). Li4 and Li30 samples were deposited at Ichthyological collection of Papanin Institute for Biology of Inland Waters, Russian Academy of Sciences, Borok, Yaroslavl, Russia. For the latter specimen, we used historical scales from scale book, a DNA source that is appropriate for mitogenome sequencing by NGS technology (Nedoluzhko et al. 2018).

DNA was isolated from caudal fin (Li4) and dried scales (Li30) in the DNA facilities of the National Research Center ‘Kurchatov institute’ (Moscow, Russia) using a standard method of DNA extraction from animal tissue (phenol-chloroform). The concentration of DNA in the samples was measured using a Qubit fluorimeter (Thermo Fisher Scientific, Waltham, MA, USA).

Two DNA libraries were prepared using an Ovation Ultralow Systems V2 kit (NuGEN, San Carlos, CA, USA). Mitochondrial genome was sequenced used Illumina Hiseq 2500 (Illumina, San Diego, CA, USA) with 150 bp pared-end reads.

33,981,829 and 144,017,525 Illumina paired-end reads were generated for DNA library of Li4 sample and Li30 sample, respectively. Illumina reads from Li30 sample were used for building mtDNA sequence de-novo by Norgal software package (Al-Nakeeb et al. 2017), but for Li4 library, there were not enough reads for building whole mitochondrial genome continuous contig. The Li4 reads were mapped to the Li30 mitochondrial genome using the Bowtie2 software version 2.2.3 with – very-sensitive-local preset options (Langmead and Salzberg 2012).

As a result, the mitogenome of *S. ischchan aestivalis* consists of 16,677 bp (GenBank accession numbers Li4: MG599465 and Li30: MG599466) and includes 13 protein coding genes (PCGs), 2 rRNA genes and 22 tRNA genes.

Eleven of the 13 PCGs (*NAD4*, *NAD5*, *NAD4L*, *NAD3*, *COB*, *NAD1*, *NAD2*, *COX2*, *ATP8*, *ATP6*, *COX3*) used ATG as start codon, another one (*COX1*) used GTG and *NAD6* used ATA. Twelve genes (*NAD1*, *NAD2*, *COX1*, *COX2*, *ATP8*, *ATP6*, *COX3*, *NAD3*, *NAD4L*, *NAD4*, *NAD5,* and *COB*) ended with a TAA stop codon, but for three of them (*COX2*, *NAD4,* and *COB*), TAA stop codon is completed by the addition of 3′ A residues to the mRNA, *NAD6* gene ended with a TAG stop codon.

**Figure 1.**
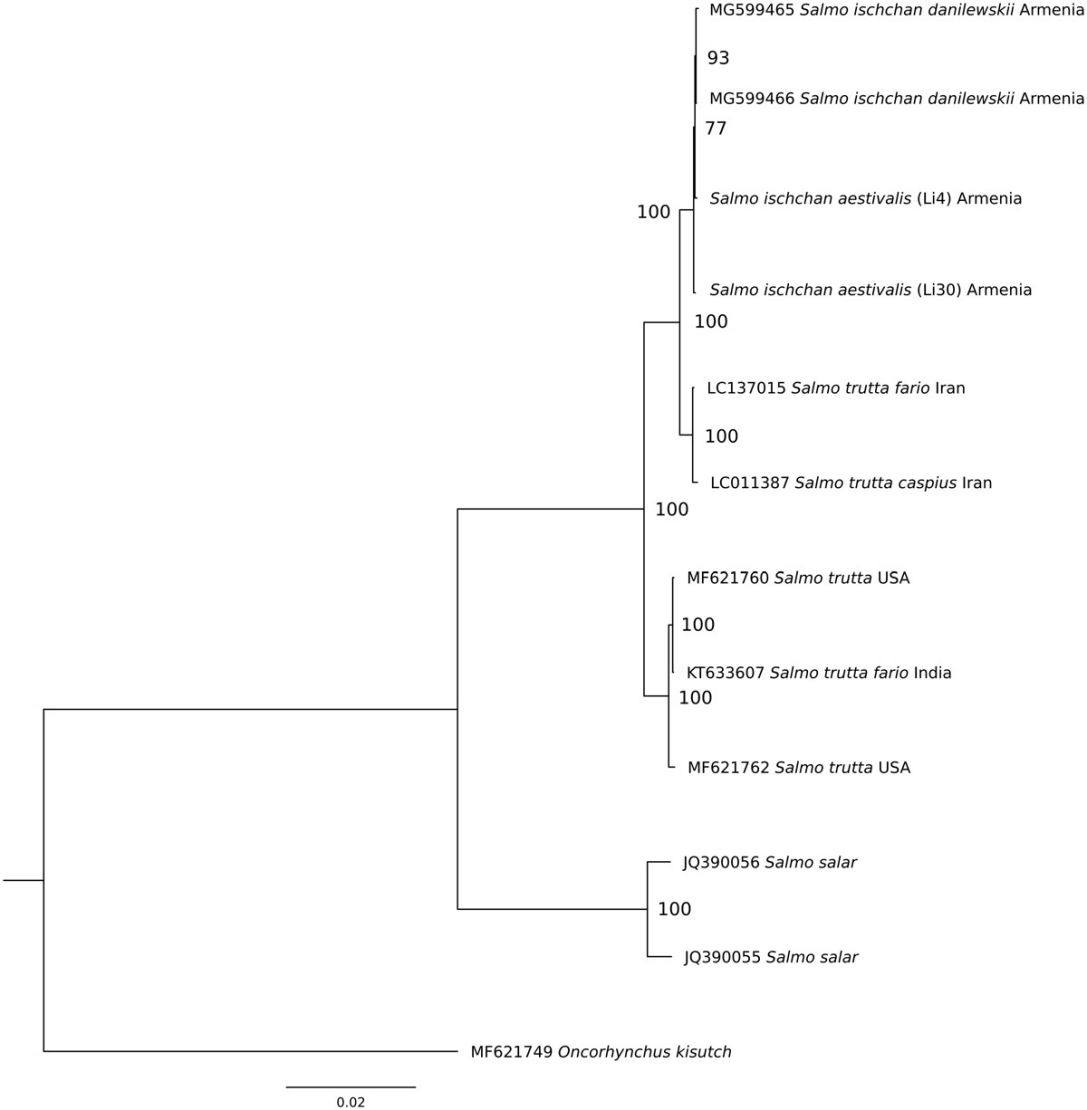
The maximum-likelihood phylogenetic tree for *Salmo ischchan aestivalis* and other salmonid species.

The phylogenetic analysis for whole mitogenome sequences was performed for the *S. ischchan aestivalis* and other Salmonidae species: *S. ischchan danilewskii* (MG599465 and MG599466), *S. trutta fario* (LC137015); *S. trutta* (MF621760); *S. trutta* (MF621762); *S. salar* (JQ390055); *S. salar* (JQ390056), and *Oncorhynchus kisutch* (MF621749) (Figure 1). Sequences were aligned using multiple sequence alignment program Muscle 3.8.31 (Edgar 2004). All gaps and poorly aligned positions were removed using Gblocks 0.91b (Talavera and Castresana 2007), resulting in 16,511 bp length alignment. The phylogenetic relationships were reconstructed using the maximum likelihood (ML) method in the PhyML 2.4.5 (Guindon and Gascuel 2003). The best substitution model (averaged for whole mitogenome) was chosen in the jModelTest 2.1.10 (Darriba et al. 2012) on the basis of the Bayesian information criterion (BIC). According to jModelTest, the best model describing the evolution of the mitogenomes was TVM + G (–lnL = 34659.93, BIC = 69611.21), and therefore, it was used for ML analysis (Figure 1).
